# Synthesis of N-Doped Graphene Quantum Dots from Cellulose and Construction of a Fluorescent Probe for 6-Mercaptopurin Quantitative Detection

**DOI:** 10.3390/ma17235852

**Published:** 2024-11-28

**Authors:** Qiang Xu, Jiayi Dong, Guiqin Yan, Rongnan Yi, Xiaojing Yang

**Affiliations:** 1 College of Environmental Science and Engineering, Shanxi University of Electronic Science and Technology, Linfen 041004, China; xuqiang@sxdzkj.edu.cn; 2 Key Laboratory of Food & Environment & Drug Monitoring and Testing of Universities in Hunan Province, Hunan Police Academy, Changsha 410138, China; 3 School of Life Science, Shanxi Normal University, Taiyuan 030006, China; cleevine@163.com (J.D.); gqyan2013@163.com (G.Y.); 4 School of Resources, Environment and Materials, Guangxi University, Nanning 530004, China

**Keywords:** cellulose, N-doped graphene quantum dots, inner filter effect of fluorescence, 6-mercaptopurine

## Abstract

With cellulose as the precursor and ethylenediamine as the N source, N-doped graphene quantum dots (N-GQDs) were synthesized by a simple and feasible one-pot hydrothermal method. The whole process did noSchemet need a strong acid or strong base and avoided interference from inorganic salt residues. The whole process lasted only 3 h and avoided any complex postprocessing. Because of the outstanding optical properties of N-GQDs, a high-efficiency 6-mercaptopurine fluorescent probe based on the inner filter effect of fluorescence was established. The detection range was 0.2–60 μM and the detection limit was 0.05 μM. This method can preliminarily detect 6-mercaptopurine in human urine and avoids any sample preparation or extraction in advance and brings satisfactory results.

## 1. Introduction

Cancers are one of the leading causes of pathopoiesis and death worldwide [1]. The decline in the total mortality rate of cancers and the increase in the total number of surviving patients reflect that significant achievements have been made in cancer prevention and cure. Nevertheless, cancers are still challenges against the persistent public health of the world. So far, chemotherapy is pivotal in therapy of various cancers. The technological development of chemotherapy has resulted in considerable revolution in cancer treatment and has greatly improved the survival rate. The total survival rate of acute lymphocyte leukemia in children is over 90% [1,2]. Such success largely depends on the daily use of 6-mercaptopurine (6-MP) for maintenance treatment. Moreover, 6-MP can be used to cure chorionic epithelioma, malignant hydatidiform mole, malignant lymphoma, and frequently occurring myeloma at the blastic phase. Hence, the demand for 6-MP is very large in clinical treatment, but too low a dose will decrease the anti-inflammatory and anti-immune effects, while too high a dose will cause nausea, vomiting, stomatitis, and diarrhea or even restrain marrows and damage livers [3]. Thus, rapid and accurate detection of 6-MP will guide clinical drug use. At present, the 6-MP detection methods include chemiluminescence analysis [4,5], electrochemical detection [6,7], high-performance liquid chromatography [8,9,10,11], mass spectrum [12], molecular imprinting [13],capillary electrophoresis [14], Raman analysis [15], and fluorescence [16,17,18]. Despite their high sensitivity and selectivity, these methods are limited by the use of expensive equipment, complex sample pretreatment, low repeatability, and tedious detection. Hence, a simple and fast 6-MP quantitative detection method with high sensitivity and selectivity is urgently needed [19].

With the development of fluorescent probes, quantum dots (QDs) are widely used in fluorescent probes thanks to their strong optical stability, long fluorescence lifetime, wide excitation peaks, narrow symmetrical emission peaks, and larger Stoke shifts. Especially, graphene quantum dots (GQDs), as a new type of fluorescent nanomaterials [20], are featured by chemical inertness, strong resistance against photobleaching, low cytotoxicity, and high biocompatibility, and thus are widely used in cell imaging, biological sensing, and drug delivery. The synthesis of GQDs has attracted the attention of many researchers. The existing synthetic methods can be divided into top–down methods and bottom–up methods. The top–down methods are mostly based on the Hummers approach [21] and adopt graphite or graphite-like carbon fibers, graphene films, or graphite powder as precursors. However, top–down methods rely on strong acids, strong bases and enhancers (e.g., potassium dichromate) for chemical oxidation stripping, and have a long preparation time, and the by-products will cause environmental pollution. The bottom–up methods are mostly based on aromatic structures (e.g., citric acid, acetylacetone, glucoses) as precursors [22] and synthesize GQDs from carbonization under microwave-, ultrasound-, or hydrothermal-assisted conditions. Despite the simple processes, special equipment is needed, and the removal of small molecules is very tedious. Moreover, the difficulty in surface modification limits the potential application of GQDs. The surface modification of GQDs by doping with other atoms not only reserves the intrinsic optical and chemical properties of GQDs, but also is favorable for wider application of GQDs. Thus, it is urgent to seek new precursors and develop a safe, simple, and low-cost synthetic strategy [20].

**Scheme 1:**
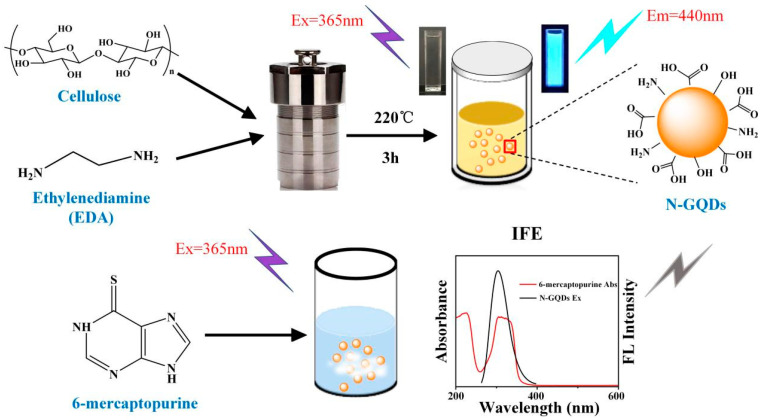
Schematic diagram of N-GQDs synthesis and 6-MP detection process.

In this study based on the green chemical method [23], N-doped GQDs (N-GQDs) were synthesized by a one-step hydrothermal method with cellulose (which is the most abundant macromolecular compound in the nature) as the precursor and ethylenediamine (EDA) as the N source. The whole process did not need a strong acid or strong base and avoided interference from inorganic salt residues and heavy metal ions. The whole process lasted only 3 h and avoided any complex postprocessing. The N-GQDs as prepared have strong photostability and salt resistance. After that, a novel high-selectivity and high-sensitivity fluorescent probe for 6-MP detection was established on the basis of the inner filter effect (IFE) of fluorescence. Moreover, it was successfully used to detect 6-MP in human urine, which confirms this N-GQDs-based fluorescent probe has high application prospects (diagram shown in Scheme 1).

## 2. Experimental Section

### 2.1. Reagents and Instruments

Cellulose was provided by Aladdin Reagent Co., Ltd. (Shanghai, China), ethylenediamine, glucose and glycine were purchased from Tianjin Komeo Chemical Reagent Co., Ltd. (Tianjin, China), lactose, D-fructose and D-galactose were purchased from Shanghai Reagent Plant No. 2 (Shanghai, China), L-tyrosine was provided by Kelon Chemical Reagent Factory (Chengdu, China), and DL-methionine was provided by Beijing Chemical Reagent Co., Ltd. (Beijing, China). Alanine, L-proline, L-cysteine, arginine, glutamic acid, and lysine were all purchased from Tianjin Guangfu Fine Chemical Research Institute (Tianjin, China). All chemicals were of analytical grade and can be used without further purification. The ultrapure water (18.2 MΩcm) used in the experiment was prepared by the German microporous ultrapure water system.

The morphology and structure of N-GQDs were characterized by a Tecnai G2 F20 electron microscope (FEI, Hillsboro, OR, USA) at an accelerating voltage of 200 kV, X-ray photoelectron spectroscopy (Thermo Scientific Nexsa, Waltham, MA, USA), and UltimaIV-185 powder X-ray diffraction (XRD). The fluorescence spectrum and resonance light scattering spectrum (RLS) of N-GQDs were recorded by a Cary Eclipse fluorescence spectrophotometer (Varian, Melbourne, Australia). The UV–visible absorption spectrum was measured by TU-1901 double-beam UV-vis spectrophotometer. Fourier transform infrared spectroscopy (FT-IR) was measured by a Varian660-IR infrared spectrometer. The fluorescence lifetime of N-GQDs was measured by an FLS1000/FS5 steady-state transient fluorescence spectrometer (Edinburgh, Livingston, UK). The concentrated solution of N-GQDs was obtained by a rotary evaporation meter (RE-2000A), and the N-GQDs powder was obtained by a freeze dryer (ZX-LGJ-1A). An AEU-200 electronic balance was used to weigh drugs.

### 2.2. Preparation of N-GQDs

In brief, 1 g of cellulose and 16 mL of ethylenediamine were added into 64 mL of ultrapure water. Then, the mixture was transferred to a 100 mL stainless steel autoclave lined with polytetrafluorethylene and hydrothermally treated in an oven at 220 °C for 3 h. After cooling to room temperature, the solution was filtrated by a 0.22 μm filter membrane and then concentrated in a vacuum rotary evaporator, followed by freeze drying to form N-GQDs powder.

### 2.3. 6-MP Detection by the N-GQDs Fluorescent Probe

Into each of the eleven 5 mL colorimetric tubes, 500 μL of phosphate buffer solution (PBS) and 100 μL of 1 mg/mL N-GQDs solution were added. Then, the 13 tubes were added with 0 (blank control), 10, 20, 30, 40, 50, 75, 100, 125, 150, 200, 250, and 300 μL of the 1 mM 6-MP solution respectively. All tubes were diluted with ultrapure water to 5 mL and placed at room temperature for 5 min, followed by measurement of FL intensity F (excitation wavelength at 340 nm). The FL intensity of the blank control was set as F_0_. Each experiment was repeated three times.

## 3. Results and Discussion

### 3.1. Characterization of GQDs

**Figure 1:**
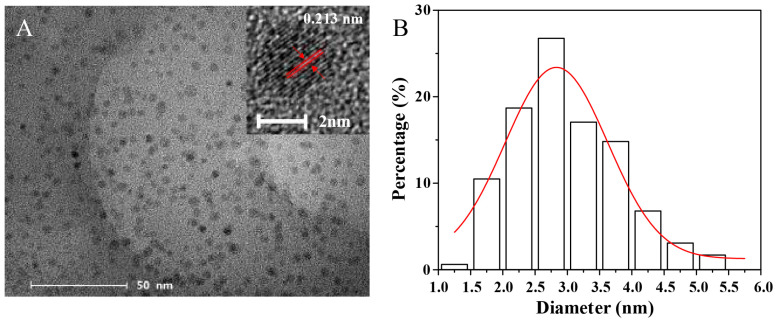
(**A**) TEM image of N-GQDs (inset: HRTEM image of one nanoparticle); (**B**) particle size distribution of N-GQDs.

Transmission electron microscopy (TEM) shows that the N-GQDs have high dispersivity and narrow particle size distribution (Figure 1A), as the particle sizes are within 1.5–5.0 nm, with a mean of 3 nm (Figure 1B). High-resolution (HR)-TEM (inset in Figure 1A) shows a clear crystal structure, with the lattice distance of 0.213 nm, which is consistent with the (100) diffraction plane of graphene. The X-ray diffraction (XRD) patterns demonstrate a broad diffraction peak at 2θ = 22.6° (Figure S1), which is attributed to the (002) diffraction of graphite, and the corresponding interlayer space is the same as that of massive graphite (0.34 nm). N-GQDs consist of a single layer or several layers of carbon atoms, which form hexagonal lattice structures through sp^2^ hybridization of carbon atoms. At the scale of quantum dots, they primarily exhibit two-dimensional characteristics and may also form three-dimensional structures through stacking or encapsulation.

**Figure 2:**
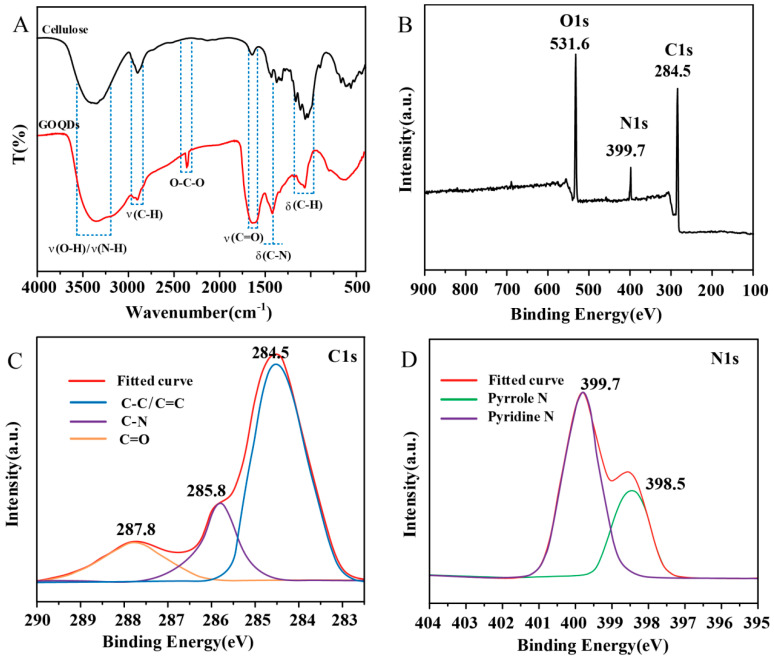
(**A**) FT-IR spectra of cellulose and N-GQDs; (**B**) XPS spectrum of N-GQDs; (**C**) C1s and (**D**) N1s high-resolution XPS spectra of N-GQDs.

Fourier transform infrared spectroscopy (FTIR) shows N-GQDs compared with cellulose demonstrate obviously stronger peaks at 2352 and 1400 cm^−1^, which correspond to the stretching vibration of O-C-O and C-N [24] (Figure 2A). Moreover, the characteristic peaks at 3421, 2931, 1643, and 1047 cm^−1^ originate from the stretching vibration of -OH, C-H, C=O, and C-H, respectively. To further study the chemical environment around the carbon and nitrogen atoms in the GQDs [25], we conducted X-ray photoelectron spectroscopy (XPS) (Figure 2B). The peaks at 284.5, 399.7, and 531.6 eV are attributed to C1s, N1s, and O1s, respectively, and the C1s spectrum (Figure 2C) demonstrates five peaks at 284.5, 285.8, 285.2, 286.8, and 288.6 eV [26,27], which are ascribed to C-C/C=C, C-N and C=O, respectively [28]. High-resolution scanning at the N1s [28] region (Figure 2D) reveals two peaks at 398.5 and 399.7 eV, which indicate the presence of pyridine N and pyrrole N [29]. FTIR and XPS both confirm that hydrothermal reactions alter the structure of cellulose, and the nitrogen atom may replace the C atom in the fibrillar structure. This doping may occur at the center or edge positions. The surfaces of N-GQDs are functionalized with multiple carboxyl and amine groups, which increases their solubility in water.

### 3.2. Optical Properties of N-GQDs

**Figure 3:**
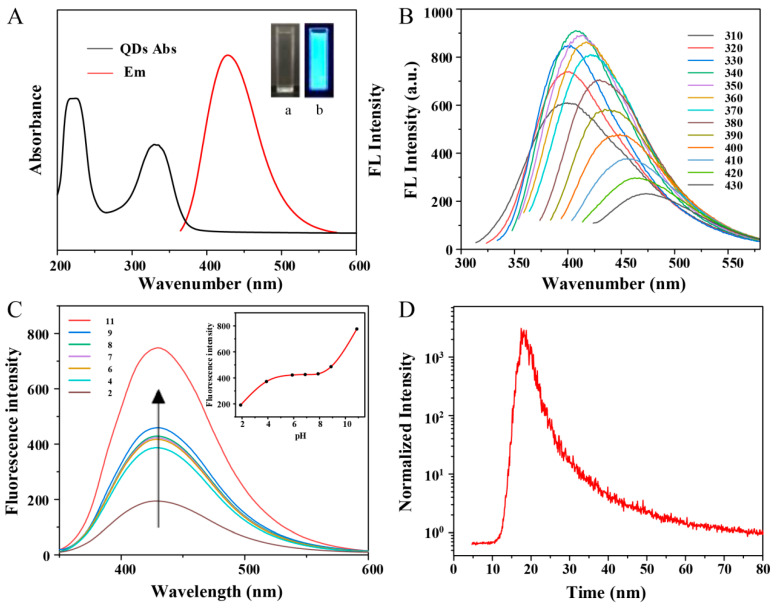
(**A**) UV-vis spectrum and fluorescence emission spectrum of N-GQDs (inset: samples under irradiation in natural light (a) and UV irradiation (365 nm, b)); (**B**) FL emission intensity of N-GQDs under excitation from 300 to 520 nm at an interval of 10 nm; (**C**) fluorescence spectra of N-GQDs dispersed in buffer solutions at different pH; (**D**) typical time-resolved fluorescence attenuation curves of N-GQDs at 440 nm (λ_ex_ = 375 nm).

The ultraviolet spectra of N-GQDs (Figure 3A) show a strong peak at 230 nm, ascribed to the π–π* transition, and a weak shoulder peak at 300 nm, corresponding to the n–π* transition [30]. The evident peak at 340 nm is the ultraviolet absorption of N-GQDs. Under excitation at 340 nm, there is an evident emission peak at 440 nm. Under natural irradiation, the QDs solution is colorless and transparent, but, in the dark, it emits obvious blue light under the fluorescence excitation at 365 nm (inset in Figure 3A). The photoluminescence (PL) spectrum of N-GQDs shows that when the excitation wavelength is prolonged from 310 to 430 nm, the fluorescence emission peak shifts from 400 to 480 nm (Figure 3B). The peak value first increases and then decreases and red shifts, which is consistent with the PL behaviors in other reports. As for biomedical applications, acidity/alkalinity is a key factor that must be considered. The FL intensity of N-GQDs changes significantly with pH [31] and is obviously quenched under acid conditions (pH > 7) and significantly enhanced under base conditions (pH > 7) (Figure 3C). The fluorescence intensity at pH = 11 is approximately 3.5 times that at pH = 2. Such an obvious response to pH is attributed to the protonation and deprotonation of the surface carboxyl group in N-GQDs. The fluorescence intensity of N-GQDs does not change obviously within pH 4 and 9, which proves that N-GQDs can basically maintain a stable fluorescence in biological environments. Under excitation at 375 nm, the emission intensity of N-GQDs decays with time (Figure 3D) and the average fluorescent lifetime of N-GQDs is 4.19 ± 0.01 ns. With quinine sulphate as a reference, the fluorescent quantum yield of N-GQDs is 30.8% under the excitation at 340 nm [21].

### 3.3. Construction of a Fluorescent Probe for 6-MP Quantitative Detection

**Figure 4:**
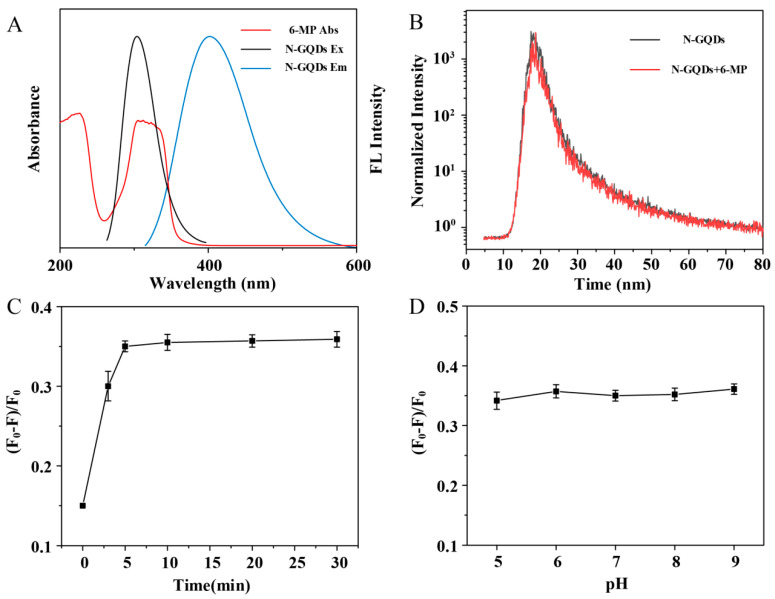
(**A**) UV-vis, excitation, and emission spectra of N-GQDs; (**B**) FL intensity decay curves of N-GQDs (λ_ex_ = 375 nm) with or without 6-MP; effects of (**C**) testing time and (**D**) acidity/basicity (pH 5–9) on the N-GQDs fluorescent probe, where F_0_ and F are the FL intensity (λ_ex_ = 340 nm) of N-GQDs without and with 6-MP, respectively.

The feasibility of N-GQDs in 6-MP detection was evaluated first. Figure S2 shows evident quenching of fluorescence of N-GQDs when 30 μM 6-MP was dripped. Hence, such effective fluorescence quenching can be utilized into 6-MP quantitative detection. On this basis, the possible detection mechanism was studied. The 6-MP shows an evident absorption peak at 345 nm (Figure 4A), which is overlapped with the excitation and emission spectra of N-GQDs. Thus, the cause for the fluorescence quenching of N-GQDs may be attributed to the inner filter effect (IFE) or fluorescence resonance energy transfer (FRET). Since FRET belongs to the dynamic quenching mechanism, such energy transfer only occurs at the excited state and will significantly alter the lifespan of fluorophores [32]. To further uncover the mechanism of fluorescence quenching of N-GQDs [33], we measured the time-resolved fluorescence spectrum of N-GQDs with the presence or absence of 6-MP. Clearly, the presence of 6-MP on the fluorescent lifetime of N-GQDs can be ignored (Figure 4B). These results validate that the fluorescence quenching of N-GQDs is a static behavior and is mainly attributed to IFE.

For the most sensitive detection of 6-MP, we optimized the testing time and pH of the N-GQDs fluorescent probe. The optimal criterion for selecting fluorescence quenching efficiency is (F_0_ − F)/F_0_, where F_0_ and F are the fluorescence intensity of N-GQDs without and with 6-MP, respectively. After the target substance is added, the testing time is a key factor of the detection system. When 6-MP (30 μM) was added into N-GQDs, the fluorescence was weakened and gradually stabilized after 5 min (Figure 4C). Hence, the reaction time between 6-MP and N-GQDs was selected to be 5 min. The effects of acidity/basicity on the fluorescent probe were studied by changing pH. The (F_0_ − F)/F_0_ was unchanged obviously within pH 5.0–9.0 (Figure 4D), indicating 6-MP can be detected within this wide range of acidity/basicity, which does not obviously interfere with the fluorescent probe.

**Figure 5:**
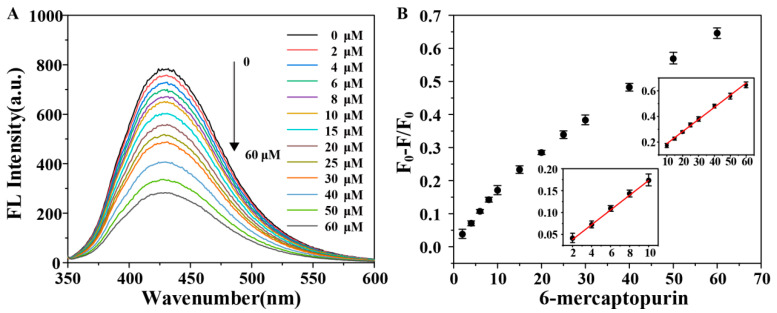
(**A**) Fluorescence emission spectra of N-GQDs (λ_ex_ = 340 nm) at different 6-MP concentrations (0, 2, 4, 6, 8, 10, 15, 20, 25, 30, 40, 50, 60 μM); (**B**) response relationship between 6-MP concentration and FL intensity of N-GQDs (inset: linear relationship between 6-MP concentration (2–10, 10–60 μM) and FL intensity of N-GQDs).

Then, the responses of the N-GQDs fluorescent probe to 6-MP were studied under the optimal conditions. As the 6-MP concentration rises, the FL intensity of N-GQDs is gradually weakened (Figure 5A). On this basis, the relation curve between fluorescence intensity and the target substance was established (Figure 5B). The linear range of N-GQDs in 6-MP detection is 0.2–60 μM, and the linear equations are y = 0.0168x + 0.0048 (R^2^ = 0.9986) and y = 0.0095x + 0.0925 (R^2^ = 0.996), and the detection limit (3σ/n) is 0.05 μM. The comparison of different 6-MP detection methods is illustrated in Table S1. Results indicate our new method is more sensitive in 6-MP detection than other methods. In comparison, our new N-GQDs fluorescent probe can be easily synthesized at low cost and without preprocessing. Moreover, the stable PL, low toxicity, and high biocompatibility of N-GQDs contribute to their huge potential in drug analysis.

### 3.4. Interference from Foreign Substances

**Figure 6:**
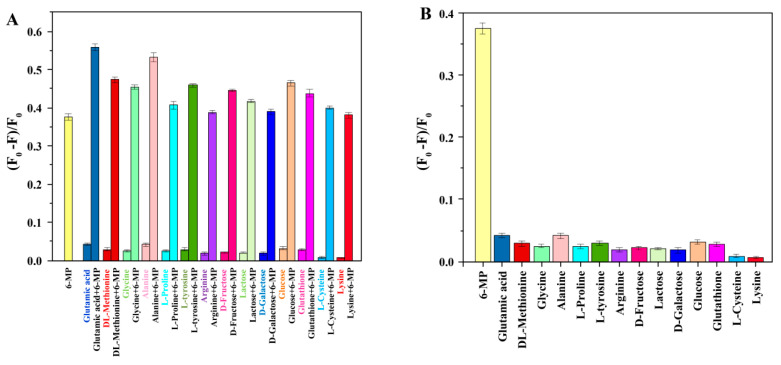
(**A**) Selective FL intensity response of N-GQDs to 6-MP (30 μM) and other potential interfering molecules (300 μM); (**B**) luminescent quenching efficiency of 6-MP (30 μM) with the presence of other potential interfering molecules (300 μM). All experiments were conducted at pH = 7.4 (λ_ex_ = 340 nm). The error bars show the standard deviations from three independent experiments.

In addition to sensitivity, the selectivity and anti-interference ability of the new fluorescent probe over the target substance in a complex real environment are also very important. Hence, several common coexisting substances (Glu, Lac, Lys, Fru, Gala, Ala, Pro, Cys, Tyr, Arg, Glu, Meth, Gly) in human body fluids were tested to validate the selectivity and anti-interference ability of the new fluorescent probe. To highlight the selectivity of the new fluorescent probe, we set the competitive substances at a 10-fold concentration (300 μM). The changes in the FL intensity of the N-GQDs fluorescent probe with the presence of competitive substances can be ignored (Figure 6A), indicating the new probe is sensitive to 6-MP to some extent. Moreover, the anti-interfering ability of the probe in response to 6-MP with the presence of other competitive substances was also detected. Even when other interfering biomolecules existed in human body fluids, the luminescent response of the probe to 6-MP was basically unchanged (Figure 6B).

### 3.5. Real Sample Detection

To validate the practicability of the new fluorescent probe, we applied it in 6-MP detection in human urine. First, urine was sampled from healthy volunteers without taking drugs. The urine samples were added with different concentrations of 6-MP and all diluted 100 times [34]. To validate the reliability and accuracy of the new method, we detected 6-MP concentrations in the real samples by using a standard addition method. The luminance intensity of the N-GQDs fluorescent probe in the spiked samples was recorded under excitation at 340 nm. The detected concentration was calculated through the established curve equation of the relationship between the sensor fluorescence intensity and the target substance concentration. The detected concentration was divided by the concentration of 6-MP added to the urine sample to obtain recovery (%). The recovery rate of urine samples was 98.05–102.68% (Table 1), indicating the new probe performs well in 6-MP detection from real samples. Compared with other potential 6-MP sensors, N-GQDs have advantages such as low production cost, nontoxicity, stable fluorescence performance, and the detection process does not require expensive detection instruments. Performance comparison of the analytical methods for the 6-MP assay shows this has a lower detection (Table S1). Hence, this new probe can be used into 6-MP detection in real human urine without any complex sample pretreatment.

## 4. Conclusions

Graphene quantum dots (GQDs) were prepared from cellulose by a one-step hydrothermal method and were surface functionalized with amino groups. This method is outstanding, with a short synthesis time and the avoidance of a strong acid, strong base, or metal impurity, and is free from interference by inorganic salt residues or complex purification of products. This method presents a new clue for the recycled use of natural cellulose and offers a new green method for the synthesis of fluorescent quantum dots from biomass. The N-GQDs have high fluorescent stability and can be used to build a 6-MP detection fluorescent probe, which was used to sensitively detect 6-MP in human urine. Importantly, this method can be potentially expanded to the detection of other drugs with an ultraviolet absorption peak at 300–400 nm. This study can expand the application of N-GQDs into analysis of various drugs.

## Figures and Tables

**Table 1:** Recovery for the determination of 6-MP concentration detection in human urine.

Samples	Spiked (Μm)	Found (μM)	Recovery (%)	R.S.D (%, n = 3)
1	2	1.97	98.50	2.31
2	6	6.09	101.50	1.07
3	10	9.89	98.90	0.97
4	20	20.41	102.05	1.31
5	40	41.07	102.68	2.07
6	60	58.91	98.18	1.01

## Data Availability

The original contributions presented in the study are included in the article and Supplementary Material, further inquiries can be directed to the corresponding authors.

## Supplementary Materials

The following supporting information can be downloaded at: https://www.mdpi.com/article/10.3390/brainsci14121214/s1, Figure S1: XRD patterns of N-GQDs; Figure S2: Fluorescence emission spectra of N-GQDs (λex= 340 nm) at 6-MP concentrations (0, 30, μM); Table S1: Performance comparison of the analytical methods for 6-MP assay; [5,6,7,11,13,18].
