# Supplementary material for: Synthesis of N-Doped Graphene Quantum Dots from Cellulose and Construction of a Fluorescent Probe for 6-Mercaptopurin Quantitative Detection
Source: Materials (Basel). 2024 Nov 28;17(23):5852. doi: 10.3390/ma17235852 (PMC11642655; doi:10.3390/ma17235852)
Supplement: Supplementary file 1 [file materials-17-05852-s001.zip › materials-3280275-supplementary.pdf]

materials

MDPI

Supplementary Materials

Synthesis of N-Doped Graphene Quantum Dots from Cellulose and Construction of a Fluorescent Probe for 6-Mercaptopurin Quantitative Detection

Qiang Xu [1,2], Jiayi Dong [3], Guiqin Yan [3], Rongnan Yi [2,*] and Xiaojing Yang [4,*]

1 College of Environmental Science and Engineering, Shanxi University of Electronic Science and Technology, Linfen 041004, China
2 Key Laboratory of Food & Environment & Drug Monitoring and Testing of Universities in Hunan Province, Hunan Police Academy, Changsha 410138, China
3 School of Life Science, Shanxi Normal University, Taiyuan 030006, China
4 School of Resources, Environment and Materials, Guangxi University, Nanning 530004, China
* Correspondence: yrn@hnu.edu.cn (R.Y.); kjcyxj@gxu.edu.cn (X.Y.)

1. The Procedure for the Determination of the Fluorescence Quantum Yields

Fluorescence (FL) quantum yields of the N-GQDs were obtained by using the comparative method. The quantum yield of N-GQDs, Φ x , is calculated according to the following equation:

$$\Phi x = \Phi\mathrm{std}\left(\frac{Fx}{F\mathrm{std}}\right)\left(\frac{A\mathrm{std}}{Ax}\right)\left(\frac{nx}{n\mathrm{std}}\right)^2$$

where Φ, F, A, and n are quantum yield of the standard sample, integrated FL intensity,absorbance, and refractive index, respectively. [1]The subscript "std" refers to the standard fluorophore of known quantum yield, for an example, quinine sulfate used in present work (The quantum yield of quinine sulfate dissolved in 0.1 M H_2SO_4 is 0.54.). To minimize re-absorption effects, the absorbance of N-GQDs and quinine sulfate solution in the 10 mm fluorescence cuvette were adjusted never exceed 0.05 at the excitation wavelength. The quinine sulfate was dissolved in 0.1 M H_2SO_4(n std was 1.33).

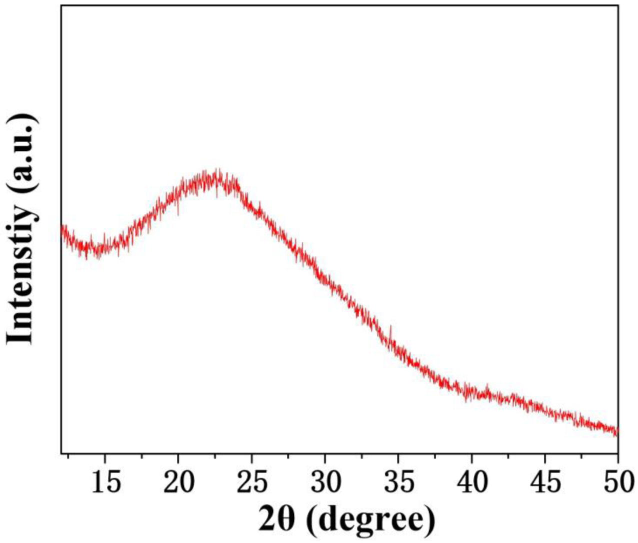

Figure S1. XRD patterns of N-GQDs.

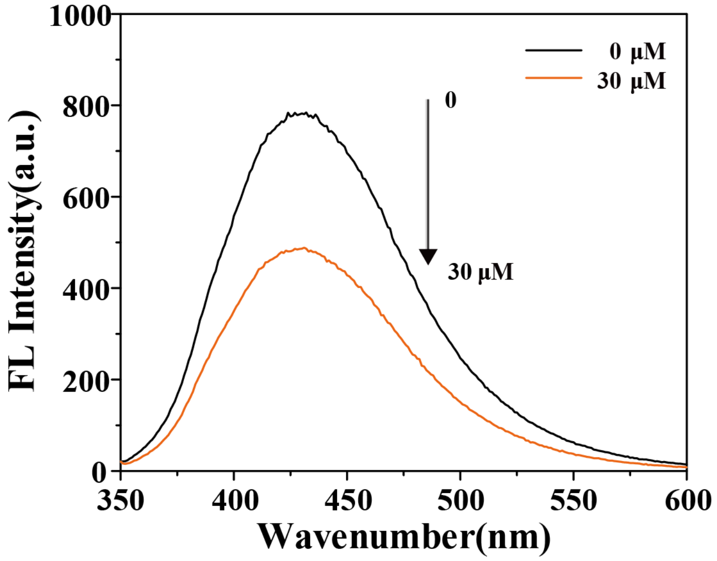

Figure S2. Fluorescence emission spectra of N-GQDs (λex= 340 nm) at 6-MP concentrations (0, 30, μM).

Table S1. Performance comparison of the analytical methods for 6-MP assay.

Analytical Methods	Preparation cos	Linear Range (μM)	Detection Limit （μM）	Ref.
MIT	Complex preparation	0.07-39.43	0.02	[1]
Chemiluminescence	Expensive preparation	0.55-55	0.16	[2]
Electrochemistry	Modification process is complex	0.2-200	0.05	[3]
Electrochemistry	Complex preparation	1-100	0.1	[4]
HPLC	Expensive equipment	0.0615-2.40	0.05	[5]
CdTe QDs	Material is toxic	0.2-3.20	0.08	[6]
GQDs	Low preparation cost and non-toxic	0.2-60	0.05	ThisWork

References

1. L. Wang, Z. Zhang, The study of oxidization fluorescence sensor with molecular imprinting polymer and its application for 6-mercaptopurine (6-MP) determination, Talanta 76 (2008) 768-771.
2. P. Biparva, S.M. Abedirad, S.Y. Kazemi, Silver nanoparticles enhanced a novel TCPO–H2O2–safranin O chemiluminescence system for determination of 6-mercaptopurine, Spectrochim. Acta A 145 (2015) 454-460.
3. P. Zhou, L. He, G. Gan, S. Ni, H. Li, W. Li, Fabrication and evaluation of [Co(phen)2L]3+-modified DNA-MWCNT and SDS-MWCNT electrodes for electrochemical detection of 6-mercaptopurine, J. Electroanal. Chem. 665 (2012) 63-69.
4. S. Shahrokhian, F. Ghorbani-Bidkorbeh, A. Mohammadi, R. Dinarvand, Electrochemical determinations of 6-mercaptopurine on the surface of a carbon nanotube-paste electrode modified with a cobalt salophen complex, J. Solid State Electrochem. 16 (2012) 1643-1650.
5. A.P. Li, J.D. Peng, M. Zhou, J. Zhang, Resonance light scattering determination of 6-mercaptopurine coupled with HPLC technique, Spectrochim. Acta A 154 (2016) 1-7.
6. M.X. Gao, J.L. Xu, Y.F. Li, C.Z. Huang, A rapid and sensitive spectrofluorometric method for 6-mercaptopurine using CdTe quantum dots, Analytical Methods 5 (2013) 673-677.

Disclaimer/Publisher's Note: The statements, opinions and data contained in all publications are solely those of the individual author(s) and contributor(s) and not of MDPI and/or the editor(s). MDPI and/or the editor(s) disclaim responsibility for any injury to people or property resulting from any ideas, methods, instructions or products referred to in the content.
